# Transition Zone1 Negatively Regulates *Arabidopsis* Aluminum Resistance Through Interaction With Aconitases

**DOI:** 10.3389/fpls.2021.827797

**Published:** 2022-01-27

**Authors:** Jiajia Liu, Benhui Shi, Mengxin Zhang, Guangchao Liu, Zhaojun Ding, Huiyu Tian

**Affiliations:** ^1^The Key Laboratory of Plant Development and Environmental Adaptation Biology, Ministry of Education, College of Life Sciences, Shandong University, Qingdao, China; ^2^Key Lab of Plant Biotechnology in Universities of Shandong Province, College of Life Science, Qingdao Agricultural University, Qingdao, China

**Keywords:** aluminum stress, root growth, TZ1, ACO, citrate

## Abstract

The soluble form of aluminum (Al) is a major constraint to crop production in acidic soils. The Al exclusion correlated with the Al-induced organic acid is considered as an important mechanism of Al resistance. The regulation of organic acid exudation in response to Al stress mediated by the root organic acid transporters has been extensively studied. However, how plants respond to Al stress through the regulation of organic acid homeostasis is not well understood. In this study, we identified the functionally unknown *Transition zone1* (*TZ1*) as an Al-inducible gene in the root transition zone, the most sensitive region to Al stress, in *Arabidopsis*. *tz1* mutants showed enhanced Al resistance and displayed greatly reduced root growth inhibition. Furthermore, TZ1 was found to interact with the aconitases (ACOs) which can catalyze the conversion from citrate, one of the most important organic acids, into isocitrate. Consistently, in *tz1* mutants, the citric acid content was highly increased. Collectively, this study provides evidence to show that TZ1 negatively regulates root growth response to Al stress through interacting with ACOs and regulating citric acid homeostasis.

## Introduction

Aluminum (Al) is a prevalent kind of metal elements in the soil and most of the aluminum is in silicate or other solid forms, which are non-toxic to plants. However, in acidic soils (pH < 5.0), the trivalent aluminum ion (Al^3+^) are released from clay minerals and are quite toxic to crop plants (Kochian et al., 2004; Chauhan et al., 2021). Al toxicity is a major limiting factor that reduces crop yields worldwide and constrains the food security, especially in the developing countries in tropical and subtropical regions abundant with acidic soils (Kochian et al., 2015). Al toxicity can target the root apex and restrain the growth of the plant root and subsequently affect the uptake of water and nutrients, which finally results in a significant reduction in crop yields (Kochian et al., 2004; Delhaize et al., 2007; Motoda et al., 2007; Xu et al., 2017).

Based on the previous studies, plants have developed two main mechanisms to deal with the Al stress: (1) Al exclusion mechanism, in which Al is prevented from entering the plant root apex and (2) Al tolerance mechanism, which involves the detoxification and sequestration of the internal Al once it enters the roots. The most widely documented Al exclusion mechanism is Al-induced release of organic compounds, specially the diverse organic acid ions, such as malate, citrate, and oxalate, to chelate toxic Al^3+^ in rhizosphere by forming non-toxic compounds and thus prevent Al^3+^ from entering the root cell (Kochian et al., 2015). Until now, numerous studies in different monocot and dicot plant species demonstrated that the members of aluminum-activated malate transporter (ALMT) and multidrug and toxin extrusion (MATE) families facilitate the Al-activated efflux of malate and citrate, respectively (Sasaki et al., 2004; Hoekenga et al., 2006; Ligaba et al., 2006; Furukawa et al., 2007; Magalhaes et al., 2007; Liu et al., 2009, 2018; Maron et al., 2010; Yang et al., 2011; Tovkach et al., 2013; Zhou et al., 2017). Based on the studies in *Arabidopsis*, ALMT1-mediated malate exudation from roots under Al stress provides a major contribution to Al detoxification (Hoekenga et al., 2006). Citrate exudation facilitated by MATE plays a minor but an important role to detoxify Al (Liu et al., 2009). Additionally, previous studies have shown that the Cys_2_-His_2_-type zinc finger transcription factor STOP1 could stimulate *ALMT1* and *MATE1* expression under Al stress (Iuchi et al., 2007; Liu et al., 2009; Wu et al., 2019; Zhang et al., 2019; Huang, 2021; Xu et al., 2021).

The tricarboxylic acid (TCA) cycle which is called citric acid cycle or the Krebs cycle in the mitochondria provides a large number of organic acids, namely malic acid, citric acid, and oxalic acid (Millar et al., 2011). The citric acid content is strictly dependent on its biosynthesis and catabolism in the Krebs cycle. Overexpressing the enzymes involved in citric or malic acid synthesis, such as citrate synthase (Barone et al., 2008; Deng et al., 2009; Han et al., 2009), malate dehydrogenase (Tesfaye et al., 2001; Wang et al., 2010), and pyruvate phosphated kinase (Trejo-Téllez et al., 2009) could all enhance the citric or malic acid synthesis and improve Al tolerance of the plant. Aconitase (ACOs) and isocitrate dehydrogenase (IDH) of the TCA cycle involved in the citrate catabolism are responsible for the adjacent catalytic reactions to convert citrate into isocitrate and then into α-ketoglutaric acid, respectively, leading to the reduction of the citric acid content. The *aco1* and *idh12* mutants showed higher accumulation of citric acid and enhanced resistance to Al stress (Anoop et al., 2003).

The root tip is the major target site of Al toxicity (Ryan and Kochian, 1993; Baluska et al., 2010; Baluska and Mancuso, 2013; Alarcon and Salguero, 2017). The transition zone (TZ) in the root tip is the boundary located between the apical meristem and the basal elongation region where the cells are in a transitional stage of cyto-architectural rearrangement, preparing to perform rapid cell elongation (Baluska et al., 2010). In addition to its role in determining the cell fate and root growth, TZ perceives endogenous hormonal signal and exogenous environmental stimuli and translates them into differential growth responses (Baluska et al., 2010; Kong et al., 2018). Studies from maize (*Zea mays*), common bean, *Arabidopsis* and sorghum indicated that TZ is the most sensitive part of the root to Al stress (Sivaguru et al., 1999; Illés et al., 2006; Rangel et al., 2007; Sivaguru et al., 2013). For example, in maize, Al treatment to the root TZ but not the elongation zone (EZ) inhibits the root growth as the effect of the treatment to the entire root apex (Kollmeier et al., 2000). Al induces ethylene production in the root TZ and subsequently alters auxin distribution in roots by disrupting AUX1- and PIN2-mediated auxin polar transport and consequently results in the root growth inhibition (Sun et al., 2010). Furthermore, ethylene signaling can enhance the auxin responses in the TZ by locally upregulating the *Trp aminotransferase* (*TAA1*) expression and results in the auxin-regulated root growth inhibition through a number of auxin response factors (ARFs) under Al stress (Yang Z. B. et al., 2014). In addition, auxin synthesis genes *YUCCAs* (*YUCs*) can be transcriptionally regulated by ethylene signaling in the root TZ in response to Al stress. EIN3 and PIF4 transcriptionally regulate Al-induced *YUC* expression and thus involve in the Al-induced auxin accumulation in root TZ and inhibit the root growth (Liu et al., 2016). Al stress triggers a cytokinin response in the root TZ, which is dependent on the ethylene signaling and inhibit the root growth (Yang Z. B. et al., 2017). Additionally, cytokinin signaling regulates the root elongation synergistically with auxin signaling in response to Al stress, which is different from their antagonistic roles at the root TZ to control the root growth without Al (Dello Ioio et al., 2008; Di Mambro et al., 2017).

To further clarify the molecular mechanism involved in plant Al resistance, we transcriptomically analyzed the gene expression profile in root tips under Al stress and identified one unknown function gene *Transition zone1* (*TZ1*), which was highly upregulated in the root TZ after Al treatment. *tz1* mutants displayed increased citrate acid content and enhanced Al resistance. Further analyses showed that TZ1 could interact with ACOs and enhance their activity to catalyze the conversion of citric acid to isocitric acid and thus regulate the Al resistance of the plant.

## Materials and Methods

### Plant Materials and Growth Conditions

The *Arabidopsis* Columbia (Col-0) and T-DNA insertion lines, such as *aco1* (*GK_138A08*), *aco2* (*SALK_090220*), and *aco3* (*SALK_014661*) were obtained from Arabidopsis Biological Resource Center (ABRC). For constructing the *pYAO*-based CRISPR/Cas9 (Yan et al., 2015) mutants *tz1-1* and *tz1-2*, two targets 5′-ATGGGAAACTGTTTGATGGG-3′ and 5′-GGGAGAAGCGGCTGCAAAGG-3′ were designed on the website http://crispr.mit.edu/. Then, *pYAO*-based CRISPR/Cas9 constructs were transformed into Col-0 plants. The *TZ1* full-length genomic sequence was constructed into pKGWFs7.1 driven by *TZ1* native promoter and transformed into Col-0 to generate the *TZ1p:TZ1-GFP-GUS* transgenic plants. Seedlings were grown on 1/2 Murashige and Skoog (MS) medium or hydroponically 2% modified molecular genetics research laboratory (MGRL) solution (pH 5.0) under a 16-h photoperiod at 22°C.

### Treatments and Experimental Conditions

In all the root growth analyses, the seeds were sown onto polypropylene mesh floating on 2% modified MGRL solution containing 0 or 6 μM AlCl_3_ (pH 5.0) for 7 days. For La^3+^ treatment, the seeds were sown onto polypropylene mesh floating on 2% modified MGRL solution containing 0 or 1 μM LaCl_3_ (pH 5.0) for 5 days. The primary root length was measured using image J software. Citric acid and Al content experiments were carried out with Al (AlCl_3_, 15 μM, pH 5.0) treatment for 24 h.

### β-Glucuronidase Staining

The 7-day-old plants growing on 1/2 MS were used for histochemical β-glucuronidase (GUS) staining experiments and the GUS staining was conducted at 37°C overnight. The staining solution contains: 0.1 M potassium phosphate buffer (pH 7.0), 2 mM potassium ferri and ferrocyanide, 0.1% Triton X-100, and 2 mM X-glucuronide. The stained plants were observed with a differential interference contrast microscope (OLYMPUS BX53, Japan).

### Citrate Content Analysis

After Al treatment, a proper amount of plant samples was weighed, added with 0.25 mol/L HCl at a ratio of 1:6 (W/V), and grounded in a centrifuge tube. The samples were bathed in water at 80°C for 20 min, with continuously shaking for several times. The supernatant was centrifuged at 12,000 g at 4°C for 20 min. The detection was conducted with the wavelength at 210 nm. The column temperature was 35°C with C18 column as the stationary phase. The mobile phase was 0.2% ammonium dihydrogen phosphate at pH 2.7, and the flow rate was 0.5.

### Aluminum Content Analysis

After treatment, the roots were washed three times with double distilled water. Then, the roots were digested with 65% ultrapure HNO_3_, and the concentration of Al was determined by graphite furnace atomic absorption spectrometer (GF-AAS, SHIMADZU, Japan).

### Hematoxylin Staining

The 7-day-old seedlings were exposed to 0 or 25 μM AlCl_3_ for 3 h and then, the roots were stained with hematoxylin according to the protocol described by Polle et al. (1978). The roots were rinsed in 5 ml distilled water for 20 min and the water was replaced with 5 ml 0.2% (w/v) hematoxylin (Merck) and 0.02% (w/v) potassium iodide solution for 15 min. Finally, the roots were re-rinsed in water. Root tips were then photographed using a digital camera (Olympus E-620).

### Confocal Microscopy Analysis

Confocal micrographs were captured using an LSM-700 device (Zeiss, Germany). To visualize the Al stress-induced expression of *TZ1p:TZ1-GFP-GUS* transgene, the 7-day-old seedlings were treated with or without Al (AlCl_3_, 25 μM, pH 5.0) for 3 h and the roots were imaged in water supplemented with propidium iodide (PI, 10 mg/L). PI and green fluorescent protein (GFP) were detected at excitation wavelengths of 488 and 561 nm, respectively.

### Subcellular Localization Assay

The *35S::TZ1-YFP* construct was generated by introducing the coding sequence (CDS) of *TZ1* into the binary vector *pX-YFP* (*p35S::X-YFP*), and then the construct was transformed into protoplasts of *Arabidopsis* mesophyll cells. The *35S::TZ1-YFP* together with the cytosolic marker ADH-RFP or plasma membrane maker ZmCRN-mCherry was transiently expressed in *Nicotiana benthamiana* leaf mesophyll cells by the infiltration of the *Agrobacterium tumefaciens* strain GV3101 carrying the corresponding plasmid. The transfected tobacco grows in the greenhouse for at least 48 h at 28°C before GFP and RFP scanning. The fluorescent signals were visualized with the LSM-700 laser scanning confocal microscope (Zeiss). The ZmCRN-mCherry and ADH-RFP signals were detected with 561 nm laser excitation and 580–675 nm emission, and the TZ1-GFP signal was detected with 561 nm laser excitation and 500–530 nm emission.

### Yeast Two-Hybrid Assay

Yeast two-hybrid (Y2H) assay was performed according to the match maker GAL4 Two-Hybrid System 3 manual of manufacturer (Clontech) (Liu et al., 2021). The CDS of *TZ1* was cloned into pENTRY and then to the plasmid *pGBKT7* by LR reaction (Takara, CA, United States). The prey TZ1-BD was used to screen a pre-transformed *Arabidopsis* root cDNA library. After culturing on synthetic medium plates (SD medium) lacking Trp, Leu, and His (SD-Trp-Leu-His) for 2 days, transformants were transferred onto SD-Trp-Leu-His-Ade. The aconitase1 (ACO1) (C-terminal) fragment was identified. Then, C-terminals of *ACO* genes were fused into *pGADT7* and the interaction with TZ1 was reconfirmed by Y2H.

### Luciferase Complementary Imaging Assay

The *TZ1* and *ACO* (C-terminal) were fused into *pCAMBIA1300-nLUC* and *pCAMBIA1300-cLUC*, respectively. Four different combinations of *A. tumefaciens* were infiltrated into the same leaves of *N. benthamiana* and cultured for 48 h (Yu et al., 2019). The leaves were treated with 0.2 mM luciferin before being detected with CCD imaging apparatus.

### Pull-Down and Western Blotting

The purified glutathione S-transferase (GST)-fused protein (ACO-GST) and maltose-binding protein (MBP)-fused protein (TZ1-MBP) were incubated with Glutathione Sepharose (GE Healthcare, catalog number 17-5132-01, IL, United States) or Amylose Resin (Biolabs, catalog number E8021S, MA, United States) overnight at 4°C. After being washed three times with buffer containing 25 mM HEPES pH 7.5, 150 mM NaCl, 1 mM DTT, the beads were boiled in sodium dodecyl sulfate–polyacrylamide gel electrophoresis (SDS-PAGE) gel loading buffer at 99°C for 10 min and subsequently western blotting was performed using anti-GST or anti-MBP antibody. Primers used for pull-down assay are listed in Supplementary Data 2. For western blotting, proteins from cell lysates were denatured and subjected to SDS-PAGE (Bio-Rad, CA, United States) and transferred to poly (vinylidene fluoride) (PVDF) membranes (Millipore, catalog number IPVH00010, MA, United States). The membranes were blocked in 1 × TBST with 5% milk for 2 h, immunoblotted with indicated antibodies at 4°C overnight, followed by incubating for 2 h with horseradish peroxidase-conjugated secondary antibody at room temperature. Blots were visualized by SuperSignal West Pico Luminol Enhancer Solution (Thermo Fisher Scientific, MA, United States).

### Co-immunoprecipitation Assay

Cells were harvested and lysed in cell lysis buffer (10 mM Tris-HCl, pH 7.5, 150 mM NaCl, 0.5 mM EDTA, 0.5% NP-40, and 1 mM PMSF) on ice for 30 min with pipetting every 10 min. Cell lysate was centrifuged and the supernatant was incubated with MYC-Trap magnetic agarose beads (Chromotek, catalog number ytma-20, Germany) at 4°C for 2 h. The beads were washed three times with dilution buffer (10 mM Tris-HCl, pH 7.5, 150 mM NaCl, and 0.5 mM EDTA) and resuspended in SDS loading buffer. The resuspended beads were boiled for 10 min at 99°C and western blotting was followed using anti-MYC (Abclonal, catalog number AE010, MA, United States) or anti-GFP (TransGen Biotech, catalog number HT801-02, Beijing, China) antibody.

### Statistical Analysis

The data between samples were compared by one way ANOVA with Student’s *t*-test. All values were presented as mean ± SD, and values of *p* < 0.05 were considered significant. * and^**^ denote differences significant at *p* < 0.05 and *p* < 0.01, respectively.

### Accession Number

Gene sequences described in this article are available in TAIR 10 under the accession numbers *TZ1* (*AT4G23870*), *ACO1* (*AT4G35830*), *ACO2* (*AT4G26970*), and *ACO3* (*AT2G05710*). The Gene Expression Omnibus (GEO) accession number for the RNA-seq data reported in this paper is GSE189946.

## Results

### *Transition Zone1* Is Involved in Aluminum Resistance

To identify new factors involved in Al resistance, especially in the root TZ, we treated the 7-day-old wild type (WT) *Arabidopsis* seedlings with or without 25μM AlCl_3_ for 3 h and performed transcriptome analyses with root tissues (approximately 0.5 cm in length from root tips). In combination with the published zone-specific gene expression profile in primary root (Brady et al., 2007; Chaiwanon and Wang, 2015), we identified 13 function unknown genes which were transcriptionally upregulated after Al treatment in the root TZ (Supplementary Data 1) and one of them is *TZ1*. To explore the biological function of TZ1, especially in response to Al stress, we generated two independent mutant lines by CRISPR/Cas9 technology and named them as *tz1-1* and *tz1-2* (Supplementary Figure 1). According to the sequencing results, both *tz1-1* and *tz1-2* are frame shift mutations.

**FIGURE 1:**
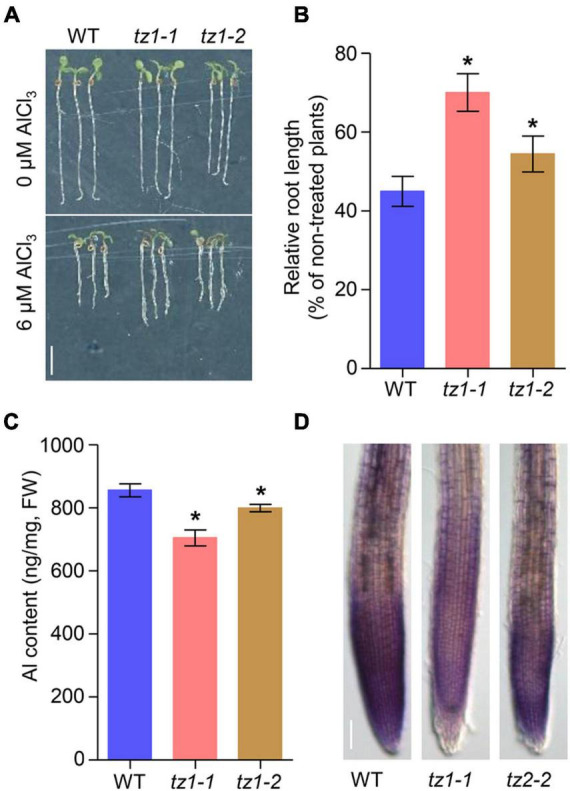
*Transition zone1* (*TZ1*) mediates the Al stress-induced inhibition of root growth. **(A,B)** Root growth of wild type (WT) and *TZ1* mutant seedlings after a 7-day exposure to 0 or 6 μM AlCl_3_. Three independent experiments were carried out, each with three replicates. Plants were grown at 22°C in long-day growth conditions. Scale bar, 0.5 cm. *p < 0.05 (Student’s *t*-test). **(C)** Al content in roots of WT seedlings and *tz1* mutants. *p < 0.05 (Student’s *t*-test). **(D)** Hematoxylin staining of root tips exposed to 25 μM AlCl_3_ for 3 h. Scale bar, 100 μm.

We then analyzed whether TZ1 is involved in Al stress response. Under normal conditions (2% MGRL solution), the two *tz1* mutants showed slightly decreased root elongation, however, after 7 days of Al treatment, the *tz1* mutants exhibited longer roots than the treated WT, displaying much less inhibited root growth (Figures 1A,B). All the results indicated that TZ1 negatively regulates Al resistance in *Arabidopsis*. To further confirm the specific function of TZ1 in Al resistance, we exposed WT and *tz1* mutants to lanthanum (La^3+^) stress and found that there was no difference in the root growth between WT and *tz1* mutants, indicating that *TZ1* responds to Al stress specifically (Supplementary Figure 2). We then measured the Al content in *tz1* mutants and WT, and the results showed less Al accumulated in the root of *tz1* mutants (Figure 1C). Moreover, the intensity of hematoxylin staining was weaker in the root tip of *tz1* mutants than that in WT, further indicating that TZ1 affects the Al accumulation in the cell wall of the root tip and thus regulates the Al resistance (Figure 1D).

### *Transition Zone1* Is Expressed in the Transition Zone and Localized in the Cytoplasm

**FIGURE 2:**
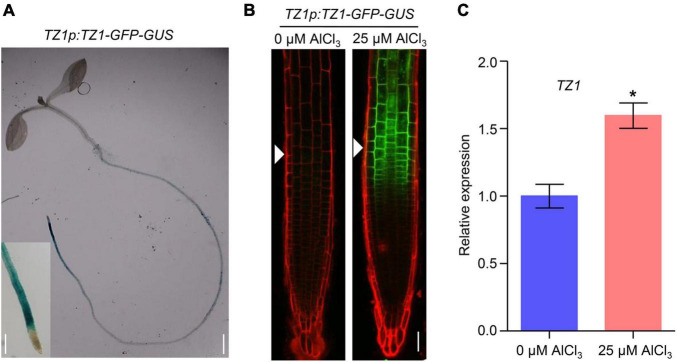
Aluminum stress-induced expression of *TZ1* in the transition zone. **(A)** The β-glucuronidase (GUS) staining of *TZ1p:TZ1-GFP-GUS* showed that *TZ1* is expressed in the *Arabidopsis* root, with the high expression in the transition zone (TZ), elongation zone (EZ), and mature zone (MZ). Left scale bar, 50 μm. Right scale bar, 1 cm. **(B)** 7-day-old *TZ1p:TZ1-GFP-GUS* seedlings were exposed to 0 or 25 μM AlCl_3_ for 3 h. Cell boundaries appear red following propidium iodide (PI) staining. The root TZ is marked with white arrowheads. Scale bar, 100 μm. **(C)** Relative expression of *TZ1* by qRT-PCR assay in the 7-day-old WT *Arabidopsis* roots treated with 0 or 25 μM AlCl_3_ for 3 h. *UBQ1* was used as the reference. Values are given as mean ± SD (*n* = 3). *p < 0.05 (Student’s *t*-test).

**FIGURE 3:**
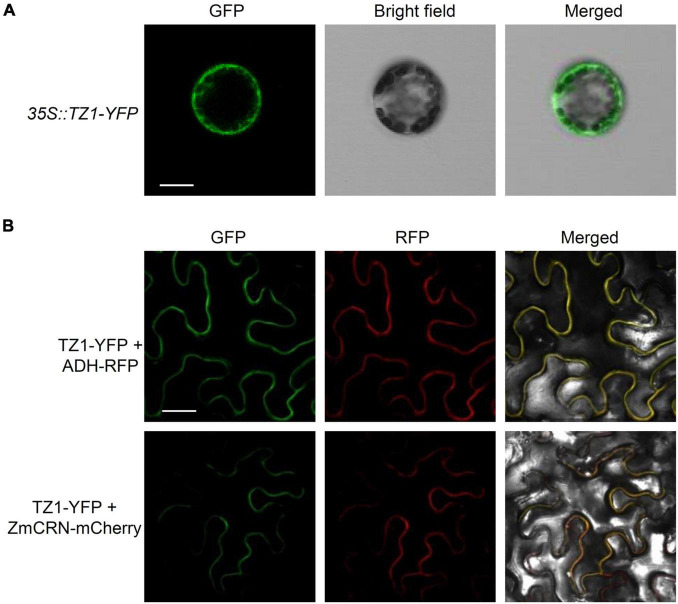
Subcellular localization of TZ1. **(A)** Plasmids containing genes encoding TZ1 protein and YFP protein were introduced into protoplasts of *Arabidopsis* mesophyll cells. Fluorescent and bright field images are shown. Scale bar, 50 μm. **(B)** Laser-scanning confocal images of TZ1-YFP fusion protein transiently expressed in *Nicotiana. benthamiana* leaf cells with ADH-RFP as a cytosolic maker or ZmCRN-mCherry as a plasma membrane maker. GFP: green fluorescent protein. RFP: red fluorescent protein. Scale bar, 50 μm.

Since TZ1 negatively regulates the Al resistance in *Arabidopsis*, we then generated *TZ1p:TZ1-GFP-GUS* transgenic plants to investigate the expression pattern of *TZ1*. GUS staining analyses showed *TZ1* is expressed in the TZ, EZ, and mature zone (MZ) of the root, with the highest expression in the root TZ (Figure 2A). Without Al treatment, GFP signal was not detected in the root tips of *TZ1p:TZ1-GFP-GUS* plants, however, after 3-h exposure to Al stress, GFP signal was highly developed in the root TZ of the transgenic plants, indicating an upregulation of *TZ1* in the root TZ (Figure 2B). Similarly, stronger GUS stain was found after Al treatment (Supplementary Figure 3). Quantitative real-time PCR (qRT-PCR) analyses of 7-day-old WT *Arabidopsis* roots with or without 25 μM AlCl_3_ treatment confirmed the increased expression of *TZ1* at transcriptional level after Al treatment (Figure 2C). It is well known that the root TZ is the most sensitive site for Al toxicity, therefore, the high expression of *TZ1* in the root TZ is consistent with the enhanced Al resistance of *tz1* mutants. Additionally, we generated *35S::TZ1-YFP* construct and transformed it into the protoplasts of *Arabidopsis* mesophyll cells to examine the subcellular localization of TZ1. The results showed that TZ1 was mainly located in the cytoplasm (Figure 3A). To further confirm the above results, we co-expressed *35S::TZ1-YFP* with the cytosolic marker ADH-RFP (Yang Y. Z. et al., 2017) and the plasma membrane maker ZmCRN-mCherry (Je et al., 2018), respectively, and found that the YFP signal of the TZ1-YFP fusion protein was completely merged with ADH-RFP, but not ZmCRN-mCherry, implying the cytosolic localization of TZ1 protein (Figure 3B).

### Transition Zone1 Interacts With Aconitases

**FIGURE 4:**
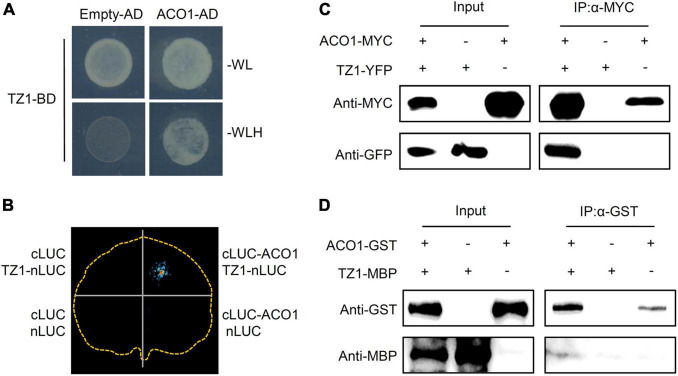
Transition zone1 interacts with ACO1. **(A)** Transition zone1 specifically interacts with aconitase1 (ACO1) in yeast. TZ1 was used as bait, the C-terminal of ACO1 was used as prey. Empty-AD was co-transformed as negative control. **(B)** Luciferase complementary imaging (LCI) analysis of interaction between TZ1 and ACO1. The N-terminal half of luciferase (nLUC) was fused to TZ1, and the C-terminal half of luciferase (cLUC) was fused to ACO1 (634–899 aa), and different construct combinations were co-expressed in *N. benthamiana*. LCI, luciferase complementation imaging. **(C)** *In vivo* Co-IP assay of TZ1 interaction with ACO1. ACO1-MYC was co-expressed with TZ1-YFP in *Arabidopsis* mesophyll cell protoplast. Protein extracts (Input) were immuno-precipitated with anti-MYC antibody (IP). Immunoblots were developed with anti-GFP antibody to detect TZ1 and with anti-MYC antibody to detect ACO1. **(D)** Western blotting assay of the GST pull down assay of ACO1-GST and TZ1-MBP.

To investigate the underlying mechanisms of TZ1 in regulating the Al resistance, we carried out Y2H experiments with the *Arabidopsis* cDNA library to screen TZ1 interacting proteins and 10 fragments were identified to interact with TZ1 (Table 1). Finally, ACO1 was selected for further analysis since ACO1 is involved in the metabolism of citric acid which is an important Al-chelator in Al resistance. In addition to the interaction of TZ1 and ACO1 in the yeast (Figure 4A), we generated ACO1-cLUC and TZ1-nLUC constructs and confirmed their interaction by luciferase complementation assay (Figure 4B). The tobacco mesophyll cells co-transformed with ACO1-cLUC and TZ1-nLUC by *A. tumefaciens*-mediated transformation showed strong LUC signal, but not the control transformed with empty vectors (Figure 4B). Moreover, TZ1-YFP was successfully detected in the anti-MYC immunoprecipitates of proteins extracted from *Arabidopsis* mesophyll protoplasts transiently expressing ACO1-MYC and TZ1-YFP (Figure 4C). Furthermore, *in vitro* pull-down assays with GST-tagged ACO1 and MBP-tagged TZ1 confirmed TZ1 interacts with ACO1 (Figure 4D). Besides *ACO1*, there are two additional ACO homologous genes in the *Arabidopsis* genome, *ACO2* and *ACO3*. Luciferase complementation and CoIP assays confirmed that TZ1 also interacts with ACO2 and ACO3 (Supplementary Figure 4).

**TABLE 1:** Yeast two hybrid (Y2H) screen for Transition zone1 (TZ1) interacting proteins.

Locus	Names	Description
AT5G60980	NTF2	RNA binding protein, mediates nuclear transport of small proteins like RanGTPase
AT4G23470	Unknown	PLAC8 family protein
AT5G25240	unknown	Stress induced protein, response to organic cyclic compound
AT5G09380	RPC4	RNA polymerase III RPC4
AT2G36530	ENO2	Involved in light-dependent cold tolerance and response to ABA in *Arabidopsis thaliana* seeds.
AT2G36120	DOT1	Encodes a glycine rich protein that is involved in leaf vascular patterning
AT4G35830	ACO1	Encodes an aconitase that participates in the TCA cycle
AT1G01010	NAC001	Response to hormone stimulus, lipid and oxygen-containing compound
AT1G08780	PFD4	Negatively regulates cold acclimation
AT5G28050	GSDA	Involved in purine nucleoside catabolic process

*Yeast two-hybrid experiments with yeast library to screen for TZ1 interacting proteins and 10 fragments were identified. Aconitase1 (ACO1) was selected for subsequent analyses.*

### The *Arabidopsis Aconitase* Mutants Display Enhanced Aluminum Resistance

**FIGURE 5:**
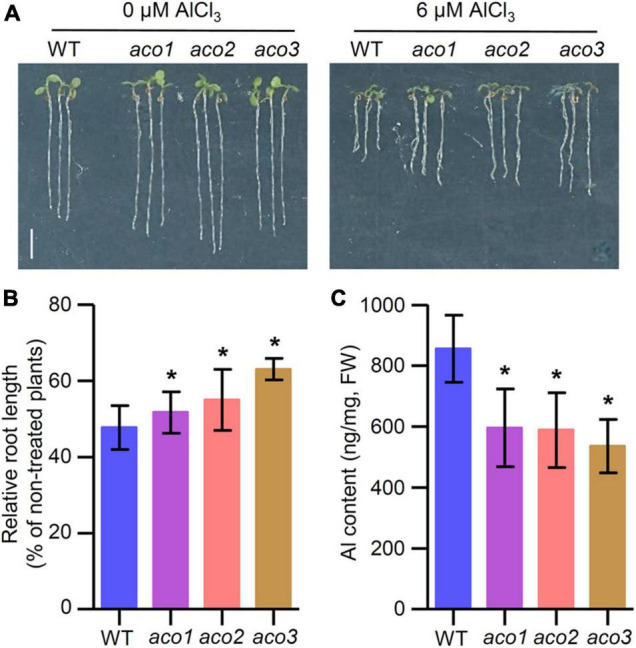
Aconitases (ACOs) mediate the Al stress-induced inhibition of root growth. **(A,B)** Root growth of WT and *aco* mutant seedlings after a 7-day exposure to 0 or 6 μM AlCl_3_. Three independent experiments were done, each with three replicates. Plants were grown at 22°C in long-day growth conditions. Scale bar, 0.5 cm. *p < 0.05 (Student’s *t*-test). **(C)** Al content in root of WT and *aco* mutants. *p < 0.05 (Student’s *t*-test).

Since TZ1 interacts with ACO proteins and *tz1* mutants display Al resistance phenotype, we tried to investigate the role of *Arabidopsis ACO* genes in response to Al stress. Under normal conditions, the root length of *aco1*, *aco2*, and *aco3* mutants exhibited slightly longer roots than WT (Figure 5A). When the plants were treated with 6 μM AlCl_3_ for 7 days, *aco1*, *aco2*, and *aco3* mutants all showed less root growth inhibition when compared with the WT control (Figures 5A,B), which is similar to the *tz1* mutants. Additionally, we detected the Al content in *aco* mutants and found that all three *aco* mutants displayed lower Al content than WT (Figure 5C). All these results implied that TZ1 may regulate the Al resistance by reducing the Al accumulation in the root through its interaction with ACO.

### Citric Acid Content Is Increased in *Transition Zone1* Mutant

**FIGURE 6:**
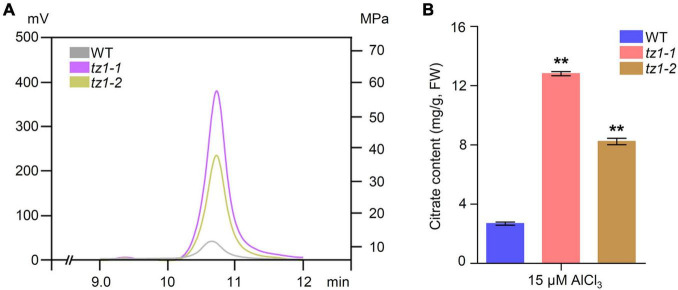
Citric acid content in *tz1.* **(A)** The citrate content in the roots were determined by high performance liquid chromatography (HPLC). The 7-day old seedings of WT and *aco* exposed to 15 μM AlCl_3_ for 24 h before measurement. **(B)** Quantification of the Al-induced citrate content in the WT and *tz1* mutants. **p < 0.01 (Student’s *t*-test).

Aconitases play crucial roles in the Krebs cycle and catalyze the conversion of citric acid to isocitric acid. It is well known that citric acid can chelate toxic Al^3+^. Therefore, we measured the citric acid content of *tz1* mutants through high performance liquid chromatography with WT plants as control. Results showed that the citric acid content of *tz1-1* and *tz1-2* mutants is higher than WT (Figures 6A,B). The higher content of citric acid is consistent with the decreased Al accumulation and increased Al resistance in *tz1* mutants, implying TZ1 may function by influencing ACO activity and finally affect the citric acid homeostasis to regulate the Al stress response.

## Discussion

Aluminum toxicity is one of the major factors that limits the crop production in acid soils (Liu et al., 2014; Sade et al., 2016). The exudation of organic acids, such as malate, citrate, and oxalate to chelate toxic Al^3+^ plays an important role in preventing Al^3+^ from entering the root cell (Kochian et al., 2015). In the current study, we characterized *TZ1* which was upregulated by Al stress in the TZ to be a negative regulator of Al resistance in *Arabidopsis* (Figures 1, 2). Furthermore, TZ1 interacts with ACOs (Figure 4 and Supplementary Figure 4) and promotes the conversion of citric acid to isocitric acid, leading to a decrease in the citric acid content and the corresponding increase in Al deposition in the root cell wall and the attenuation of root growth in Al stress response.

Due to the crucial role of citric acid in Al resistance, its secretion and accumulation in response to the Al stress have been extensively studied (Liu et al., 2018; Wang et al., 2018; Qiu et al., 2019; Matonyei et al., 2020). The Al-activated root citrate exudation is facilitated by the citrate transporter MATE (Sasaki et al., 2004; Hoekenga et al., 2006; Ligaba et al., 2006; Furukawa et al., 2007; Magalhaes et al., 2007; Liu et al., 2009, 2018; Maron et al., 2010; Yang et al., 2011; Tovkach et al., 2013). The transcription factor STOP1 is well known to upregulate the *MATE* expression and thus controls the release of citrate (Iuchi et al., 2007; Sawaki et al., 2009; Fang et al., 2020). The Al resistance of plants could be achieved by increasing the *in vivo* citrate content. Citrate is an intermediate of tricarboxylic acid cycle and glyoxylate cycle and its content was controlled by the level of the relevant enzymes involved in citrate metabolism. The *aco1*- and *idh12* mutants with more citrate displayed increased levels of Al resistance (Anoop et al., 2003). Overexpressing CS in tobacco, papaya, and *Arabidopsis* all lead to higher resistance to Al toxicity (De la Fuente et al., 1997; Koyama et al., 2000; Deng et al., 2009; Han et al., 2009). Our study first found an functionally unknown protein TZ1 interacts with ACOs (Figure 4 and Supplementary Figure 4) to negatively regulate the Al resistance by interfering with the citrate accumulation (Figure 6).

The activities and level of the citrate metabolism related enzymes are regulated by Al stress. It was reported that the activity of citrate synthases (CS) could be enhanced by Al stress in rye and Yuzu (*Citrus Junos* Sieb. ex Tanaka) (Li et al., 2000; Deng et al., 2009). Al stress affects the gene expression of citrate metabolism related enzymes including the upregulation of *CS* and *ACO1* that catalyze the conversion of citric acid to isocitric acid in the TCA cycle and the downregulation of isocitrate dehydrogenase1 (*IDH1*) (Anoop et al., 2003). In our study, TZ1, an ACOs-interacting protein, is induced by Al stress at transcriptional level (Figure 2), presumably enhancing the activity of ACOs through the directly interaction with ACOs. Under Al stress, the interaction with TZ1 enhances the activity of ACO and thus balances the overproduced citrate by Al-activated citrate synthase. It is interesting to clarify how the activity of ACOs is affected by the interaction with TZ1. Our present results showed that TZ1 interacts with the C-terminal of ACOs (Figure 4 and Supplementary Figure 4). Whether the interaction with TZ1 influences the binding of ACOs to the substrate citrate, or the conformation and activity of ACOs, requires more research in the future.

**FIGURE 7:**
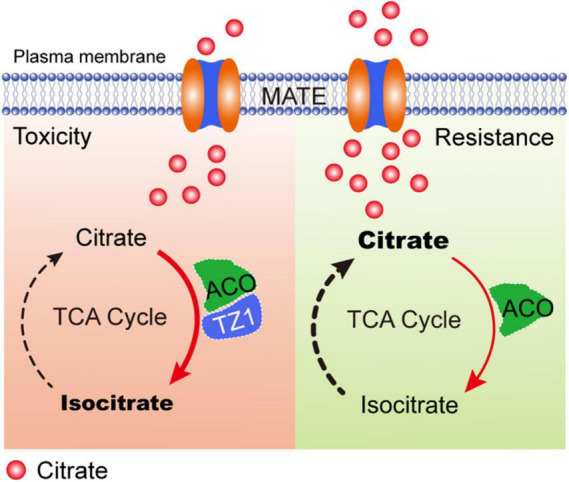
A proposed model of TZ1 and ACO mediated Al-stress response to control root growth. In toxicity responses, TZ1 is induced by Al stress and the interaction with ACO enhances the conversion of citrate to isocitrate and slows down the plant growth. Under Al resistance condition, the decreased TZ1 releases ACO and thus reduces the activity of ACO, which finally results in the high citrate content and enhanced Al resistance and plant growth.

Previous studies have shown that the root TZ is the most sensitive site in response to Al stress. Multiple phytohormones, such as auxin, cytokinin, and ethylene interact with each other in the root TZ and are involved in the regulation of root growth under Al stress (Dello Ioio et al., 2008; Sun et al., 2010; Yang Z. B. et al., 2014; Liu et al., 2016; Di Mambro et al., 2017; Yang Z. B. et al., 2017). However, little is known about whether the root TZ is an important site for other factors, such as organic acids in response to Al toxicity. In this study, *TZ1* is highly upregulated in the root TZ after Al treatment and regulates plant Al resistance *via* ACO-regulated citrate catabolism (Figure 7). In Al toxicity responses, with the increased citrate production by CS, TZ1 is induced and interacts with ACOs to enhance the activity of ACO and finally promotes the conversion of citrate to isocitrate, and slow-down the plant growth. Under Al resistance condition, the decreased TZ1 releases ACO and results in the reduced activity of ACO, which finally results in the high content of citrate and increased Al resistance and plant growth. However, whether this proposed model of TZ1-ACOs module is widespread in nature requires further investigations in the future.

## Data Availability Statement

The datasets presented in this study can be found in online repositories. The names of the repository/repositories and accession number(s) can be found in the article/Supplementary Material.

## Author Contributions

HT, ZD, and JL planned and designed the experiments. JL, BS, MZ, and GL performed the major experiments. HT and JL wrote the manuscript. All authors contributed to the article and approved the submitted version.

## Conflict of Interest

The authors declare that the research was conducted in the absence of any commercial or financial relationships that could be construed as a potential conflict of interest.

## Publisher’s Note

All claims expressed in this article are solely those of the authors and do not necessarily represent those of their affiliated organizations, or those of the publisher, the editors and the reviewers. Any product that may be evaluated in this article, or claim that may be made by its manufacturer, is not guaranteed or endorsed by the publisher.
