# Supplementary material for: Transition Zone1 Negatively Regulates Arabidopsis Aluminum Resistance Through Interaction With Aconitases
Source: Front Plant Sci. 2022 Jan 27;12:827797. doi: 10.3389/fpls.2021.827797 (PMC8829429; doi:10.3389/fpls.2021.827797)
Supplement: Supplementary file 1 [file Data_Sheet_1.PDF]

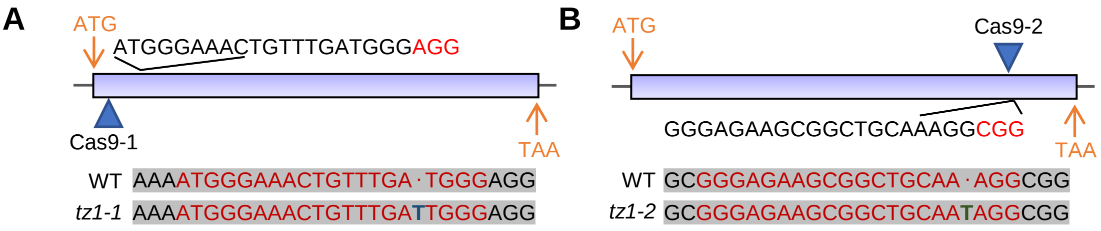

Supplementary Figure 1. Schematic representations of the *TZ1* mutant

(A) and **(B)** Schematic representations of the *TZ1* genomic sequence. The target sequences in the *TZ1* gene in front of the PAM site NGG is on the exon. The upper row shows a genomic DNA of the *TZ1*, and the lower row shows the target sequence.

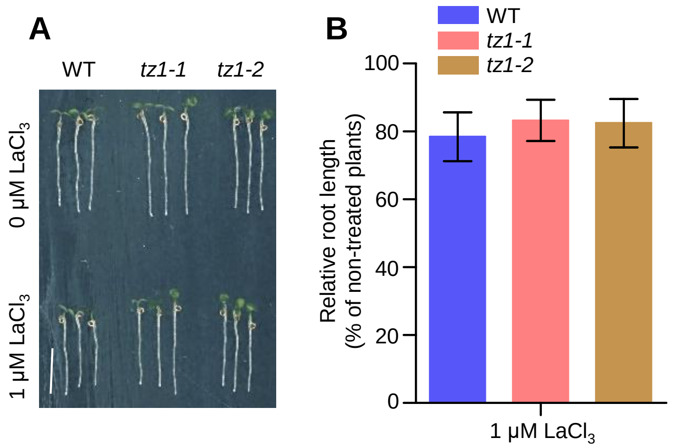

Supplementary Figure 2. TZ1 does not respond to La stress

(A) and **(B)** Root growth of WT and *tz1* mutant seedlings after 5-day exposure to 0 or 1 μM $LaCl_3$. Plants were grown at 22℃ in long-day growth conditions. Scale bar, 0.5 cm.

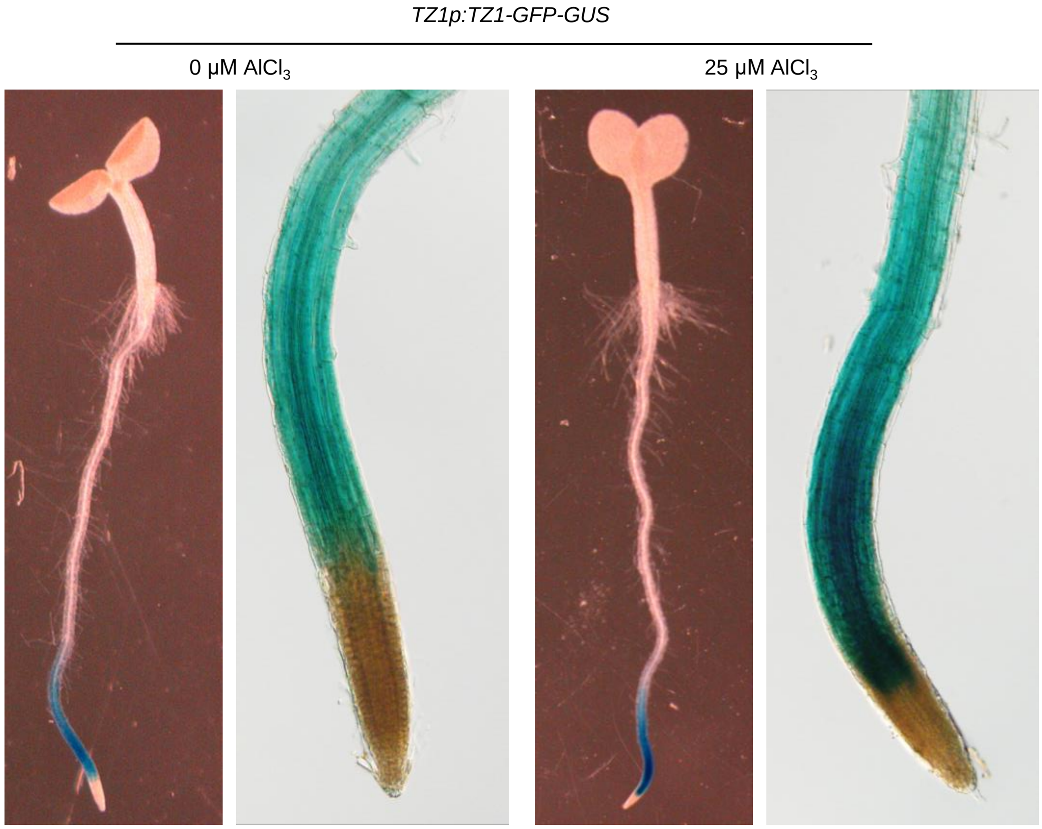

Supplementary Figure 3. Al stress induces TZ1 expression.
6-day-old *TZ1p:TZ1-GFP-GUS* seedlings were exposed to 0 or 25 μM $AlCl_3$ for 3 h. The GUS staining of *TZ1p:TZ1-GFP-GUS* showed that TZ1 is induced by Al stress.

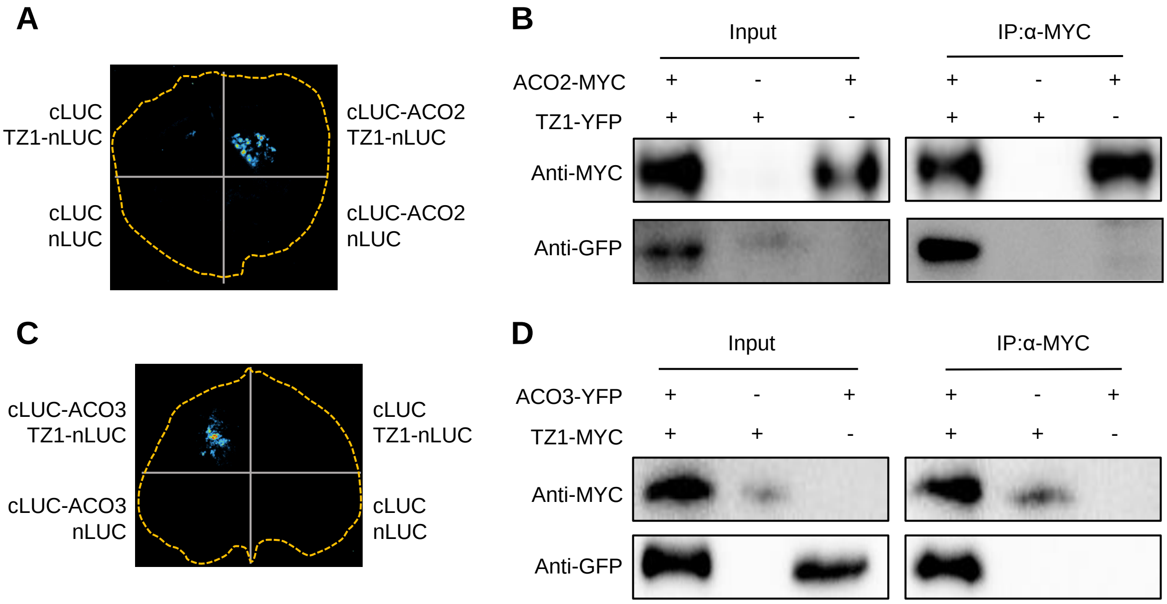

Supplementary Figure 4. TZ1 interacts with ACO2 and ACO3.

(A) LCI analysis of interaction between TZ1 and ACO2. The N-terminal half of luciferase (nLUC) was fused to TZ1, and the C-terminal half of luciferase (cLUC) was fused to ACO2(735-996), and different construct combinations were coexpressed in *N. benthamiana*. LCI, luciferase complementation imaging.

(B) In vivo Co-IP assay of TZ1 interaction with ACO2. ACO2-MYC was co-expressed with TZ1-YFP in *Arabidopsis* mesophyll protoplast. Protein extracts (Input) were immuno-precipitated with anti-MYC antibody (IP). Immunoblots were developed with anti-GFP antibody to detect TZ1 and with anti-MYC antibody to detect ACO2.

(C) LCI analysis of interaction between TZ1 and ACO3. The N-terminal half of luciferase (nLUC) was fused to TZ1, and the C-terminal half of luciferase (cLUC) was fused to ACO3(726-991), and different construct combinations were coexpressed in *N. benthamiana*. LCI, luciferase complementation imaging.

(D) In vivo Co-IP assay of TZ1 interaction with ACO3. ACO3-YFP was co-expressed with in *Arabidopsis* mesophyll protoplast. Protein extracts (Input) were immuno-precipitated with anti-MYC antibody (IP). Immunoblots were developed with anti-GFP antibody to detect ACO3 and with anti-MYC antibody to detect TZ1.
